# Television Viewing Time in Hong Kong Adult Population: Associations with Body Mass Index and Obesity

**DOI:** 10.1371/journal.pone.0085440

**Published:** 2014-01-10

**Authors:** Yao Jie Xie, Sunita M. Stewart, Tai Hing Lam, Kasisomayajula Viswanath, Sophia S. Chan

**Affiliations:** 1 School of Public Health, The University of Hong Kong, Hong Kong SAR, China; 2 Department of Psychiatry, University of Texas Southwestern Medical Center at Dallas, Dallas, Texas, United States of America; 3 Center for Community-Based Research, Dana-Farber Cancer Institute/Department of Society and Behavioral Sciences, Harvard School of Public Health, Boston, Massachusetts, United States of America; 4 School of Nursing, The University of Hong Kong, Hong Kong SAR, China; University of Chieti, Italy

## Abstract

**Background:**

Obesity is increasing dramatically in the Asia-Pacific region particularly China. The population of Hong Kong was exposed to modernization far earlier than the rest of China, reflecting conditions that are likely to be replicated as other Chinese cities undergo rapid change. This study examined the relationship between television viewing and obesity in a Hong Kong sample. Information about the relationship between a key sedentary behavior, TV viewing, and obesity, and its moderation by demographic characteristics may identify sectors of the population at highest risk for excess weight.

**Methods:**

Data were from Hong Kong Family and Health Information Trends Survey (2009–2010), a population-based survey on the public's use of media for health information and family communication by telephone interviews with 3,016 Hong Kong adults (age≥18 years). TV viewing time, body mass index (BMI), physical activity and other lifestyle variables were analyzed.

**Results:**

Viewing time was longer in women, increased with age but decreased with education level and vigorous physical activity (all P<0.01). Longer TV viewing time was significantly associated with higher BMI (Coefficients B = 0.17, 95% CI: 0.11, 0.24) after adjusting for age, gender, employment status, marital status, education level, smoking activity and vigorous physical activity. This association was stronger in women than men (Coefficients B: 0.19 versus 0.15) and strongest in those aged 18 to 34 years (Coefficients B = 0.35). Furthermore, an hour increase in daily TV viewing was associated with 10% greater odds of being obese.

**Conclusions:**

A significant socioeconomic gradient in television viewing time was observed. TV viewing time positively associated with BMI and obesity. The TV viewing – BMI associations were strongest in women and young adults, suggesting vulnerable groups to target for obesity prevention by decreasing TV viewing.

## Introduction

Obesity has become a worldwide epidemic. Among the leading risk factors for mortality in the world, overweight and obesity are responsible for 5% of deaths globally [1], and nearly 3 million deaths every year worldwide [2]. Mean body mass index (BMI) levels are increasing rapidly in the Asia-Pacific region [3], especially in China [4]. Contributors to obesity include increased availability of processed foods and lower physical activity (PA) [3]. A sedentary lifestyle, including extended time watching TV, has also been identified to be significantly associated with elevated risk of obesity and other diseases [5], [6]. Sedentary activities such as time spent seated has been reported to be much higher in Hong Kong than in most western countries [7], and Mainland China [7]. One fifth of the world's population lives in China, Hong Kong is the most westernized and urbanized city of China and its lifestyle conditions can forewarn that are likely to soon prevail in many parts of China as the later undergo rapid socioeconomic developments. This study examined the relationship between TV viewing and obesity in a Hong Kong sample. Information relevant to the relationship between sedentary behavior and obesity in Hong Kong can help to develop health promotion initiatives, and to target obesity prevention for high risk groups in China and other rapidly developing countries.

As an indicator of sedentary behavior, television viewing is the most commonly reported daily activity during leisure time [8]. Several studies have reported that prolonged TV viewing is associated with increased BMI or obesity during different stages of the life course from childhood [9]–[11], adolescence [12], [13] to adulthood [6], [14]–[16]. However, some contradictory results show little association between obesity and TV viewing [17], [18]. Diverse lifestyle and behavior characteristics in different populations may lead to different results.

TV viewing patterns are influenced by individual characteristics such as gender, age, education level and occupation [19], [20]. Vulnerable subgroups who could most benefit from interventions to prevent obesity may be identified by examining the characteristics of people who watch TV and whether these factors moderate the association between TV viewing and BMI/obesity. The possible association between TV viewing and BMI/obesity and the strength of association, particularly in relation to individual characteristics, has not been investigated in observational studies in Hong Kong and Mainland Chinese adult populations. The present study aimed: 1) to describe the TV viewing characteristics of different demographic groups of adults in Hong Kong; 2) to examine the association of TV viewing time with BMI and obesity, and 3) to identify the demographic and lifestyle characteristics of the subgroups with the strongest association between TV viewing and BMI/obesity.

## Methods

### Data sources

We analyzed the data from the Hong Kong Family and Health Information Trends Survey (HK-FHINTS) in 2009 and 2010. HK-FHINTS is an urban and representative general population health information and family communication survey of the Hong Kong civilian, non-institutionalized adult population. This survey is one of the components under the Hong Kong FAMILY project, which is funded by the Hong Kong Jockey Club Charities Trust as an initiative to promote family health, happiness and harmony. The survey is conducted annually to monitor the public opinion and perception towards family health, happiness, harmony, family communication and health information seeking behaviors. The study protocol was approved by the Institutional Review Board of the University of Hong Kong and Hospital Authority Hong Kong West Cluster.

The survey was administered by the Hong Kong Public Opinion Program of the University of Hong Kong (HKPOP). Telephone surveys were completed by trained interviewers using random-digit dialing. These interviewers were experienced in telephone surveys of the general population using the Computer-Assisted Telephone Interview System [21]. The target population was Cantonese-speaking Hong Kong residents aged 18 years or above. In a successfully contacted household, one qualified member of the household was selected by using the “next birthday” rule. Interviewers explained the study to participants. Oral informed consent was obtained from the participants before administering the questionnaire. Participants had the right to refuse answering any questions at any time without any consequences. Agreement to complete the questionnaire was considered consent. The institutional review board waived the need for written consent from the participants as this was not possible for telephone survey. A total of 1510 and 1506 respondents successfully completed the interview in 2009 and 2010 respectively. The contact rate was defined as the proportion of all calls made in which a responsible member of the housing unit was reached by the interviewers. The response rate was defined as the proportion of all completed interviews in the total number of eligible reporting units in the sample. The numerator was the number of completed interviews, and the denominator included the number of non-interviews (non-contacts/refusal/break-off/others) plus the number of interviews (complete interviews/partial interviews) [22]. The contact rate and response rate in 2009 were 28.7% and 78.8% respectively. These rates in 2010 were 28.5% and 66.4% respectively. The present analysis thus had a final sample size of 3,016, with the total response rate of 72.6%. The gender, age and district of residence of the respondents were similar to Hong Kong census population data. Cohen's effects were small at 0.02 to 0.17, which suggest that the sample was quite representative.

### Measures

#### Assessment of BMI and overweight/obesity

Respondents reported their height and body weight in the telephone interview. BMI was calculated as body weight in kilograms divided by height in meters squared (BMI = kg/m^2^). BMI was analyzed as a continuous and as a categorical variable. Using the World Health Organization standard for Asian populations [23], respondents were categorized as underweight (BMI<18.5 kg/m^2^), normal weight (18.5 kg/m^2^≤BMI<23.0 kg/m^2^), overweight (23.0 kg/m^2^≤BMI<25.0 kg/m^2^) and obese (BMI≥25 kg/m^2^). The previous three categories were designated as non-obese (BMI<25.0 kg/m^2^) in some analyses to compare with the obese group (BMI≥25 kg/m^2^) [6].

#### Assessment of TV viewing time and PA

Respondents reported the average time they spent watching television on a typical day (minutes or hours/day). Minutes were converted to hour(s) and recorded to the nearest 0.1 hour. Respondents also provided information about the duration and frequency of two types of PA, namely, vigorous PA and moderate PA. They reported the number of days that they spent for at least 10 minutes of vigorous PA in the past seven days, and the time that they usually spent on vigorous PA on one of those days. Vigorous PA was defined as those activities that made one breathe much harder than normal, such as aerobics, heavy lifting, or fast bicycling. Moderate PA was defined as the activities that made one breathe somewhat harder than normal. Some examples of this activity are bicycling at a regular pace or carrying light loads. The total time spent in moderate-to-vigorous physical activity (MVPA) was calculated as the sum of these two activities, according to physical activity guidelines for health benefits [24]. Insufficient MVPA and sufficient MVPA were then defined by <150 min/week and ≥150 min/week respectively [24].

#### Assessment of socio-demographic and lifestyle variables

Information about the following variables was also derived from the telephone interview: gender (male or female), age (recorded in 10-year groups), employment status (working hours per week), marital status (never married, currently married, and other), educational level (primary or below, secondary education, tertiary education, university degree or above), smoking (currently smoking or not) and alcohol drinking (currently drinking or not).

### Statistical analysis

Participants provided name, contact number and other demographic information in the two separate surveys, and we did not find any repetition by identifying these information. Each year of raw data were weighted for gender and age using Hong Kong population figures obtained from the government Census and Statistics Department for each year. As respondents in the two surveys showed no significant differences in demographic characteristics, TV viewing time and obesity (all P>0.05), the combined weighted data were pooled for all analyses. This method is consistent with that used in other similar surveys [21].

To address the first aim, one-way ANOVA was conducted to compare TV viewing time by different demographic and lifestyle factors, and by BMI categories. For variables with more than two categories, post-hoc comparisons were used to examine the difference between groups. Before testing the second aim which examined the association between TV viewing and BMI, analyses were conducted to determine which demographic characteristics and lifestyle factors had significant associations with BMI and therefore were potential confounding factors. One way ANOVA and Pearson correlations were used for categorical and continuous variables, respectively. Those variables that were significantly associated with BMI were adjusted in the linear regression models.

To address the second aim, multiple linear regression models were applied to examine the association of TV viewing time with BMI, which were both used as continuous variables. BMI was regressed on TV viewing time, controlling for potential confounding factors. In the next step we tested the third aim, i.e. moderation of the association between TV viewing time and BMI by demographic characteristics and lifestyle factors. Interaction terms were constructed for each potential moderator with TV viewing and tested individually. For those variables that were shown to be moderators, stratified models were constructed to elucidate the direction of moderation.

As a part of the second aim, we also examined whether individuals who were obese (BMI≥25 kg/m^2^) had different associations with TV viewing than the non-obese (BMI<25 kg/m^2^). Binary logistic regression analyses were performed to calculate odds ratios (ORs) and 95% confidence intervals (CIs) for being obese per hour increase in daily TV viewing time. Moderation was tested as above. Furthermore, we did subsample analysis with the underweight (BMI<18.5 kg/m^2^) excluded, to see whether the relationship between TV and obesity was different from the overall sample. Statistical analyses were performed in SPSS 19.0 (SPSS Institute). Findings were considered significant when P<0.05.

## Results

Females comprised 54% of the 3016 respondents. The mean age was 45.4 (standard deviation, SD = 17.2) years. Nearly seventy percent were married (67.8%), more than half were employed (54.3%), and more than one quarter had university degree or above (27.0%). Only 9% were current smokers and 10.8% drank alcoholic drinks at least once per week. The mean BMI was 22.5 (SD = 3.4) kg/m^2^. The prevalence of obesity (BMI≥25 kg/m^2^) was 25.6% in men and 15.2% in women. The overall prevalence was 20.0%.

**Figure 1:**
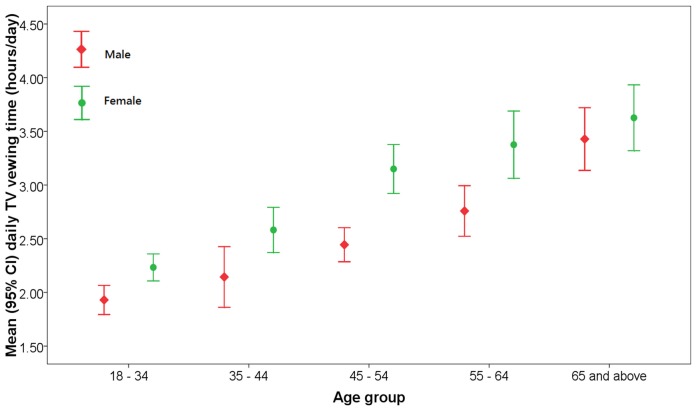
Mean hours of TV viewing daily by age and gender in Hong Kong, 2009–2010. Weighted to the total Hong Kong 2009 and 2010 population, respectively.

Table 1 and Figure 1 show that women watched TV half an hour longer on average per day (2.9 versus 2.4 hours/day) than men. Viewing time increased with older age, decreasing education level and greater BMI categories and longer in unemployed and married respondents. Those who engaged in vigorous physical activities during the previous 7 days spent less time watching TV (2.8 versus 2.5 hours/day), but no significant association was found between TV viewing time and moderate physical activities.

**Table 1:** TV viewing time (hours/day) in different demographic groups.

	N	Television viewing (hours/day) mean (SD)	P ^a^
**Gender**			<0.01
Female	1618	2.9 (2.1)	
Male	1385	2.4 (1.8)	
**Age, years**			<0.01
18–34	869	2.1 (1.4)**	
35–44	590	2.4 (2.1)**	
45–54	652	2.8 (1.9)**	
55–64	431	3.1 (2.1)**	
65+	462	3.5 (2.3)**	
**Employment status**			<0.01
Employed	1636	2.1 (1.4)	
Unemployed	1368	3.3 (2.4)	
**Marital status**			<0.01
Never married	968	2.3 (1.7)	
Currently Married and other	2036	2.9 (2.0)	
**Education level**			<0.01
Primary or below	463	3.5 (2.5)**	
Secondary education	1476	2.9 (2.0)**	
Tertiary education	251	2.2 (1.1)**	
University degree or above	813	2.0 (1.0)**	
**BMI, kg/m^2^**			<0.01
<18.5 (underweight)	248	2.1 (1.4)**	
18.5-<23.0 (normal weight)	1437	2.5 (1.8)**	
23.0-<25.0 (overweight)	554	2.9 (2.2)**	
= or >25.0 (obesity)	557	3.0 (2.3)**	
**Alcohol drinking**			<0.01
Not currently drinking	1937	2.8 (2.1)	
Currently drinking	1038	2.4 (1.8)	
**Smoking activity ^b^**			0.72
Not currently smoking	2729	2.7 (1.9)	
Currently smoking	271	2.7 (2.4)	
**Vigorous physical activity ^c^**			<0.01
No	1716	2.8 (2.0)	
Yes	1286	2.5 (1.9)	
**Moderate physical activity ^d^**			0.44
No	1323	2.7 (2.0)	
Yes	1678	2.6 (1.9)	
**Moderate-to-vigorous physical activity**			0.36
<150 minutes/week	1477	2.7 (1.9)	
= or >150 minutes/week	1511	2.6 (2.0)	

a. P values were generated from one-way ANOVA.

b. There were no significant differences in TV viewing time between ex-smokers and non-smokers, or between ex-smokers and current smokers. The ex-smokers and non-smokers were categorized to “not currently smoking”.

c. During the last 7 days, did you have at least 10 minutes of vigorous physical activities?

d. During the last 7 days, did you have at least 10 minutes of moderate physical activities?

post hoc comparisons were used, all group differences are significant (P<0.01).

Simple linear regression analysis showed a positive association between TV viewing time and BMI. The crude regression coefficient was 0.21 (95% CI: 0.15, 0.28; P<0.001). Because gender, age, employment status, marital status, education level, and vigorous PA were significantly associated with BMI and TV viewing time (all P<0.05), they were included as control variables in the multiple linear regression model for the relationship between TV viewing time and BMI. As shown in Table 2, after adjusting for age and gender, the regression coefficient decreased slightly to 0.17, but it did not change after further adjustment for employment status, marital status, education level and vigorous PA. This finding indicated that BMI increased by 0.17 kg/m^2^ (95% CI: 0.11, 0.24; P<0.001) for every hour increase in TV viewing time. There was a significant interaction between TV viewing time and gender on BMI, and also between TV viewing time and age on BMI. The stratified models showed a stronger association in women than men (coefficients B: 0.18 versus 0.14). The strongest coefficient was observed in individuals aged 18 to 34 years (coefficients B = 0.34). No significant interactions of TV viewing with smoking, alcohol drinking, vigorous PA, educational level, employment status or marital status on BMI were found (all P>0.05, data not shown).

**Table 2:** Linear regression analyses predicting body mass index (BMI) from daily TV viewing time.

Model	N	Coefficients B	SE	P
**Simple regression model ^a^**				
TV viewing	2795	0.21	0.03	<0.001
R = 0.123				
**Multiple regression model 1 ^b^**				
TV viewing	2795	0.17	0.03	<0.001
R = 0.280				
**Multiple regression model 2 ^c^**				
TV viewing	2795	0.17	0.03	<0.001
R = 0.307				
**Multiple regression model 3 ^d^**				
TV viewing	2795	0.17	0.03	<0.001
R = 0.314				
**Interaction examination models ^e^**				
TV viewing * gender	2795	0.15	0.06	0.02
R = 0.317				
R square change = 0.002				
TV viewing * age	2795	−0.14	0.07	0.03
R = 0.316				
R square change = 0.001				
**Stratified models by gender ^f^**				
Female	1507	0.19	0.04	<0.001
Male	1289	0.15	0.05	0.01
**Stratified models by age ^g^, years**				
18–34	780	0.35	0.09	<0.001
35–44	556	0.14	0.07	0.04
45–54	629	0.13	0.07	0.08
55–64	411	0.22	0.08	0.01
65+	420	0.08	0.07	0.22

a. Only TV viewing time entered the model as independent variable.

b. Model was adjusted for gender and age.

c. Model was adjusted for gender, age, employment status, marital status, education level.

d. Model was adjusted for gender, age, employment status, marital status, education level and vigorous physical activity.

e. Models were adjusted for age, employment status, marital status, education level and vigorous physical activity. The interaction terms were constructed for each moderator.

f. Models were adjusted for age, employment status, marital status, education level and vigorous physical activity.

g. Models were adjusted for gender, employment status, marital status, education level and vigorous physical activity.

Logistic regression analysis indicated that the crude odds of being obese was 11% greater with each hour of time spent watching TV (OR: 1.11, 95% CI: 1.06, 1.15, P<0.001). Gender, age, employment status, marital status, education level, alcohol drinking and vigorous physical activity were significantly associated with obesity and TV viewing time (all P<0.05), and after adjusting for these variables, the odds ratio reduced slightly to 1.10 (95% CI: 1.05, 1.15, P<0.001) (Table 3). There was a significant interaction between TV viewing time and age on obesity (P<0.01), and the OR was the greatest in respondents aged 18–34 years (OR: 1.38, 95% CI: 1.19, 1.61, P<0.001). No significant interaction between TV viewing and gender on obesity, and between TV viewing and other demographic and lifestyle factors on obesity was observed (all P>0.05, data not shown). The results after excluding the underweight participants showed a similar pattern: the similar overall odds ratio of being obese and a significant interaction between TV viewing and age, and the greatest odds ratio in respondents aged 18–34 years (data not shown).

**Table 3:** Odds ratios (ORs) for obesity per hour increase in daily TV watching time by logistic regression analysis.

Model	N	OR	95% CI		P
			Lower Bound	Upper Bound	
**Overall model ^a^**					
TV viewing	2772	1.10	1.05	1.15	<0.001
**Stratified models by gender^ b^**					
Female	1727	1.08	1.02	1.16	0.02
Male	1045	1.11	1.04	1.19	<0.01
**Stratified models by age^ c^, years**					
18–34	659	1.38	1.19	1.61	<0.001
35–44	379	1.15	1.04	1.28	<0.01
45–54	712	1.06	0.95	1.18	0.32
55–64	631	1.10	0.99	1.23	0.07
65+	391	0.98	0.88	1.08	0.66

a. Multiple logistic regression analysis was used to examine the association of overweight/obesity with TV viewing. Model was adjusted for gender, age, employment status, marital status, education level, alcohol drinking and vigorous physical activity.

b. Models were adjusted for age, employment status, marital status, education level, alcohol drinking and vigorous physical activity.

c. Models were adjusted for gender, employment status, marital status, education level, alcohol drinking and vigorous physical activity.

## Discussion

We found a significant socioeconomic gradient in television viewing time in Hong Kong adults. Prolonged TV viewing time was significantly associated with BMI. This association was strongest in women and young adults. Our finding is consistent with a previous study [19] that indicated that prolonged TV time is associated with the indices of social disadvantage and older age. Younger people with higher levels of education may be more likely to be employed in occupations that provide an extensive social network with more social support [20], [25]. Thus, this group may spend more time with friends or family members for social gatherings that displace the time spent watching television [6], [26]. These results indicate that offering the less educated, older and unemployed people with enjoyable alternatives for relaxation during leisure time (e.g., yoga, tai chi) could be a beneficial strategy to help decrease TV viewing time among the socio-economically disadvantaged people.

Consistent with most previous studies [6], [13]–[16], [27], [28], TV viewing time was positively associated with BMI/obesity. One proposed explanation is that the time spent for watching television displaces time spent in PA [17], [29]. Individuals who spend more time watching TV tend to have lower energy expenditure [15]. The energy expended in watching TV is lower than that in other sedentary activities [30]. However, some other studies showed that TV viewing was not closely related to PA [6], [31]. In our study, individuals with longer TV viewing time had less vigorous PA, but no significant association was found with moderate PA. Furthermore, consistent with previous studies [6], [19], [26], [32], [33], the positive association between TV viewing time and BMI/obesity was independent of PA. Findings from these studies suggest that the mechanisms of developing obesity from TV viewing are beyond simple decreases in PA.

As a mass medium, television is influential because of its extensive reach and the cumulative effects of exposure to media messages over time [29]. Evidence indicates a positive relationship between TV viewing and overall increased food intake [9], [34], [35], coupled with the fact that people tend to follow an unhealthy eating pattern during TV viewing [26]. Moreover, greater exposure to television advertising leads individuals to choose nutrient-poor but energy-dense foods [36], [37]. In addition to direct effects of TV viewing on obesity, indirect contributions have also been suggested, such as cooking shows that do not consider food preparation as a practical skill but are promoted as a type of entertainment [38]. An absence of concern about the long term consequences of weight gain may also exist because of unrealistic beliefs about the ease of weight reduction among those exposed to reality television shows in which contestants lose large amount of weight in brief periods of time [39]. Therefore, it is possible that reduced TV viewing time could result in lower energy intake as well as increased energy expenditure through more low-intensity activity.

In our study, TV viewing interacted with gender and age on its effects on BMI. The association between TV viewing and BMI was stronger in women and individuals aged 18 to 34 years. Logistical regression analysis confirmed a significant interaction between age and TV viewing on obesity. This result raises the question on whether metabolic health is affected differentially by TV viewing in women and young adults. A large, population-based study [40] in Australia showed that the association between TV viewing and cardio-metabolic biomarkers was stronger in women than in men [5], [31], [40]. Women have a higher average fat mass and a lower average skeletal muscle mass than men. This finding suggests that women are more susceptible to the negative impacts of sitting for a long time, typically when they have consumed a large evening meal [41]. A significant interaction between TV viewing and gender on BMI was found in our study. However, this interaction did not appear in the logistic regression analysis because the odds of being obese in women were not significantly different from men. This result indicates that TV viewing for women was more strongly associated with greater weight over the whole range of weight than for men. However, a smaller difference was observed between genders, whether TV viewing was associated with obesity or not. This finding demonstrates that gender moderation is applicable when the relationship was linear. However, we suggest that women need more attention for obesity prevention because they spend more time watching TV. A longitudinal study [16] that examined the changes of TV viewing time from adolescents to young adults suggested that individuals who reduced their TV viewing time from adolescents to young adulthood were at less risk of becoming obese, whereas individuals who continued or increased their TV viewing time until young adulthood were at a greater risk of increased BMI and obesity. Our findings further show that young people (aged 18 to 34 years) who had prolonged TV viewing time were more likely to be obese compared with older people. Early life behaviors may predict later health outcomes in life [42], and spending more time watching TV during youth has been identified as a strong predictor of adverse health outcomes later in life [38]. Thus, young adulthood is an important period for obesity prevention. More effective ways to reduce TV viewing in women and young adults are needed, which may include encouraging them to go out for more PA or engage in other healthy activities during leisure time.

Some limitations of the present study need to be acknowledged. First, the cross-sectional design cannot confirm causality or clarify the direction of relationships. Secondly, a previous study [43] that examined the association between screen-time and obesity showed a steady increase in the risk of obesity with increasing screen-time. Furthermore, computer and video time may vary in different social-demographic groups. Those with high education level (who are also more likely to be employed) are likely to have increased computer screen time and decreased TV viewing time. In our study, respondents with higher education levels had shorter TV viewing time (table 1), and reported more physical activity. It is possible that higher activity levels compensated for other screen time, as their BMI was lower than those with low education levels (university degree or above: 21.8±3.1 kg/m^2^; below university degree: 22.7±3.5 kg/m^2^, P<0.01). Nevertheless, a limitation of our study is that information about other screen-time including video game time and computer screen time was not available, and might have confounded our findings. In addition, we did not obtain information about physical disability in old age, which could attenuate the relationship of obesity with screen-time [43]. Further studies are needed to examine the complex interrelationships among sitting time, physical disability and obesity in older age. Finally, self-report measures are subject to random and systemic error. Recall difficulties or impaired recall, such as under reporting of some variables (e.g., TV viewing time, PA and weight), socially desirable responses, or other reporting biases can potentially affect the results. A prospective evaluation of changes in factors over time, and the addition of objective measures for the key variables including TV viewing, PA and BMI, should enhance our understanding of how demographic and lifestyle factors interact with TV viewing behavior in the development of obesity. These findings can inform appropriate interventions tailored to the needs of different subgroups in Hong Kong and other populations [44].

Despite these limitations, to our knowledge, there are no other comparable studies to evaluate the association between TV viewing and BMI/obesity in Hong Kong and Mainland Chinese adult populations. Socio-cultural changes towards modernization in Hong Kong have preceded those in China Mainland, and our findings may serve as an indicator of the effects that may become apparent in many parts of China and other rapidly developing countries. Our findings highlight that women and young adults are particularly susceptible to gaining weight from prolonged TV viewing. Further studies are required to clarify the mechanism of TV viewing on obesity among susceptible populations, as well as to develop effective approaches to reduce TV viewing time and its related obesity especially in high risk populations.
